# Mice as paratenic hosts of *Aelurostrongylus abstrusus*

**DOI:** 10.1186/s13071-019-3293-2

**Published:** 2019-01-22

**Authors:** Vito Colella, Martin Knaus, Olimpia Lai, Carlo Cantile, Francesca Abramo, Steffen Rehbein, Domenico Otranto

**Affiliations:** 1 Dipartimento di Medicina Veterinaria, Università degli Studi di Bari, Str. prov. per Casamassima km 3, 70010 Valenzano (Bari), Italy; 2 Boehringer Ingelheim Vetmedica GmbH, Kathrinenhof Research Center, Walchenseestr. 8–12, 83101 Rohrdorf, Germany; 3 Department of Veterinary Sciences, University of Pisa, Viale delle Piagge 2, 56124 Pisa, Italy

**Keywords:** Feline, Cats, Paratenic host, Rodents, Mice, Epidemiology, Lungworm

## Abstract

**Background:**

Several species of nematodes included in the superfamily Metastrongyloidea are recognized agents of parasitic infections in felines*. Aelurostrongylus abstrusus* is the most prevalent species affecting the respiratory system of domestic cats. The route of infection in cats is supposed to be through ingestion of gastropod intermediate or paratenic hosts. However, because gastropods are not the preferred preys of cats, rodents were suggested to play an important role as paratenic hosts in the biological cycle of *A. abstrusus* and in the epidemiology of aelurostrongylosis.

**Results:**

Two studies were conducted to document histopathological tissue lesions in mice experimentally infected with *A. abstrusus* third-stage larvae (L3) (Study 1), and to determine larval counts in their organs (Study 2). Additionally, cats were fed with experimentally infected mice to assess their infectivity. *Aelurostrongylus abstrusus* L3 were recovered from the liver, spleen, brain, skeletal muscle and gastrointestinal tract tissues by artificial digestion, and heart, spleen and brain tested positive for *A. abstrusus* at molecular diagnosis. Multifocal encephalitis and meningitis and glial nodules were the most common histopathological lesions found in mice inoculated with *A. abstrusus*. All cats shed first-stage larvae of *A. abstrusus* after ingestion of mice inoculated with this nematode.

**Conclusions:**

In this study, we provide information on the anatomical localization, histopathological alterations and rate of recovery of *A. abstrusus* L3 in mice, and confirm their infectivity to cats (definitive hosts) after feeding on infected mice (paratenic hosts). Data presented here add knowledge to further understand the biology of *A. abstrusus* in mice and underline the importance of mice as paratenic hosts of this nematode for the infection of cats.

## Background

Infection with several species of nematodes of the superfamily Metastrongyloidea may trigger cardiopulmonary clinical signs in feline hosts [1–3]. While some of them, such as *Troglostrongylus brevior* [4, 5] and *Angiostrongylus chabaudi* [6] have only recently been found to affect domestic cats, *Aelurostrongylus abstrusus* has been historically designated as the cat lungworm [7]. These metastrongyloids parasitize both domestic and wild felids, and their life-cycles involve gastropods (snails and slugs) as intermediate hosts (IHs), often coexisting in the same ecological niches [7, 8]. A recent large-scale survey in Europe demonstrated that 11% of domestic cats are infected with at least one species of lungworm, mainly *A. abstrusus* and *T. brevior*, or co-infected by lungworm species [2].

The clinical and pathological presentation of feline aelurostrongylosis is mainly related to the inflammatory reaction caused by immature stages and to the localization of the adult nematodes in the respiratory system (i.e. bronchioles and alveolar ducts) [9]. Transmission of *A. abstrusus* to its definitive hosts may occur through the ingestion of gastropods, but it is thought to be mainly due to the predation of vertebrates that serve as paratenic hosts (PHs) [7]. In addition, shedding of infective third-stage larvae (L3) into the environment by gastropods [10] and snail-to-snail transmission, referred to as intermediesis [11], have been proposed as alternative routes of transmission for feline lungworms in IH populations. However, although lacking confirmatory data, there is an alleged emetic effect caused by the ingestion of molluscs [4], and gastropods are not the prey of choice in domestic cats’ dietary habits [12]. Thus, the attitude of cats towards the predation of potential PHs (i.e. rodents, birds, reptiles and frogs) are likely of importance in the transmission and the maintenance of the life-cycle of the parasite [7, 13–16].

In the early 20th century, mice were erroneously indicated as intermediate hosts of *A. abstrusus* [17], and further studies attempted to understand the role of mice as PHs [15, 18, 19]. Nevertheless, limited data is available on the anatomical localization of *A. abstrusus* L3 in mice and their infectivity to cats after ingestion of infected rodents.

This study provides information on the anatomical localization, the histopathology, and the rate of recovery of *A. abstrusus* L3 in experimentally infected mice, and on the infectivity for cats of *A. abstrusus* after ingestion of mice previously inoculated with different *A. abstrusus* larval doses.

## Results

### Study 1

No gross lesions were observed at necropsy of mice infected with 30 *A. abstrusus* L3 and necropsied 10, 20, 30, 45 and 60 days post-inoculation (dpi) (two mice per time point) and mice which served as uninfected controls.

Examination of mice infected with 30 *A. abstrusus* L3 allowed for the recovery of single L3 from the liver, spleen and brain by artificial digestion, and heart, spleen and brain tested positive for *A. abstrusus* DNA (Table 1). Histopathological examination of the brain, spleen, liver, kidneys, lungs and diaphragm, however, revealed microscopical changes of variable extent, onset and duration of occurrence relative to the time point of inoculation, with the brain as the organ which most often showed histopathological changes (Table 2).

**Table 1:** Study 1. Results of examination of organs of mice inoculated with ~30 *A. abstrusus* third-stage larvae each at intervals after inoculation (two mice per time point) by artificial digestion and for detection of *A. abstrusus* DNA

Mouse	Days post-inoculation of ~30 *A. abstrusus* third-stage larvae									
	10		20		30		45		60	
	AD	PCR	AD	PCR	AD	PCR	AD	PCR	AD	PCR
	1	2	1	2	1	2	1	2	1	2
Brain	0	-	1	-	1	-	0	-	0	+
Diaphragm	0	-	0	-	0	-	0	-	0	-
Heart	0	+	0	-	0	-	0	-	0	-
Kidneys	0	-	0	-	0	-	0	-	0	-
Liver	1	-	0	-	0	-	0	-	0	-
Lungs	0	-	0	-	0	-	0	-	0	-
Spleen	1	+	0	-	0	-	0	-	1	-
Skeletal muscle	0	-	0	-	0	-	0	-	0	-

*Abbreviations*: *AD* artificial digestion: actual number of *A. abstrusus* third-stage larvae recovered from half of an organ’s digest, *PCR* detection of *A. abstrusus* DNA by PCR; +, PCR-positive, -, PCR-negative

**Table 2:** Study 1. Frequency of major histopathological changes recorded in the organs of mice inoculated with ~30 *A. abstrusus* third-stage larvae each by histological examination of half organs at intervals after inoculation (two mice per time point)

Days after inoculation	Number of mice demonstrating histopathological alterations in the								
	Brain			Spleen	Liver	Kidneys	Lungs	Diaphragm	
	Multifocal encephalitis with perivascular cuffing of small lymphocytes	Multifocal lymphocytic encephalitis	Glial nodules	Multifocal extramedullar haematopoiesis	Extramedullar haematopoiesis	Multifocal mononuclear cell infiltration of cortical and medullary interstitium	Multifocal lymphocytic peribronchial inflitration	Thickening of serous membrane through mixed cell infiltration	Lymphocytic diaphragmitis
10	2/2	2/2	0/2	1/2	1/2	1/2	1/2	0/2	0/2
20	2/2	2/2	1/2	1/2	1/2	1/2	2/2	0/2	0/2
30	2/2	2/2	1/2	1/2	2/2	1/2	2/2	1/2	1/2
45	1/2	1/2	2/2	0/2	0/2	0/2	0/2	1/2	1/2
60	2/2	2/2	2/2	0/2	0/2	0/2	0/2	2/2	2/2

**Fig. 1:**
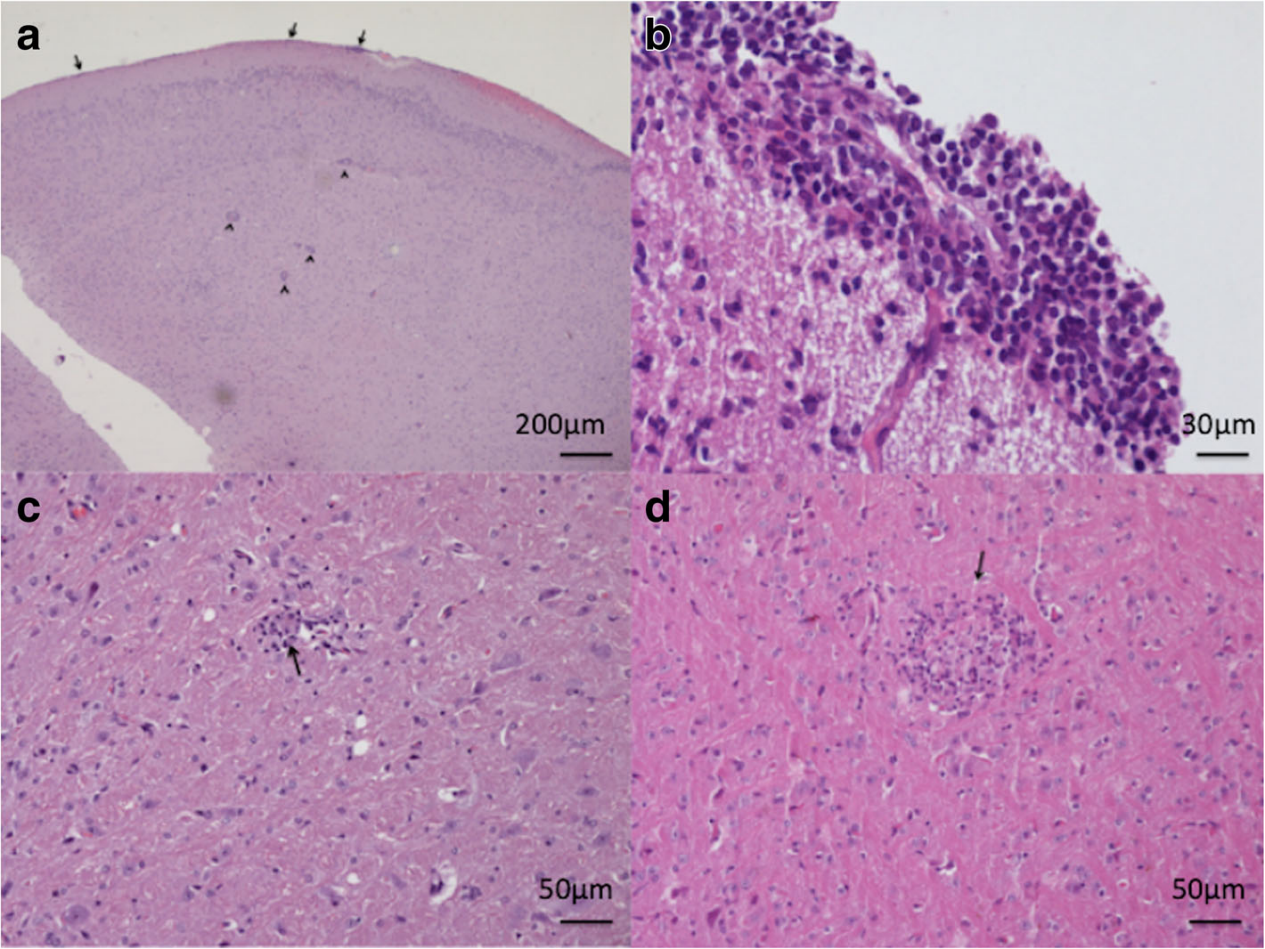
Study 1. Brain. **a** Meningitis (arrows) and encephalitis with lymphocytic perivascular cuffing (arrowhead). **b** Lymphocytic meningitis. **c** Small glial nodule (arrow). **d** Large glial nodule (arrow). *Scale-bars*: **a**, 200 μm; **b**, 30 μm, **c**, **d**, 50 μm

**Fig. 2:**
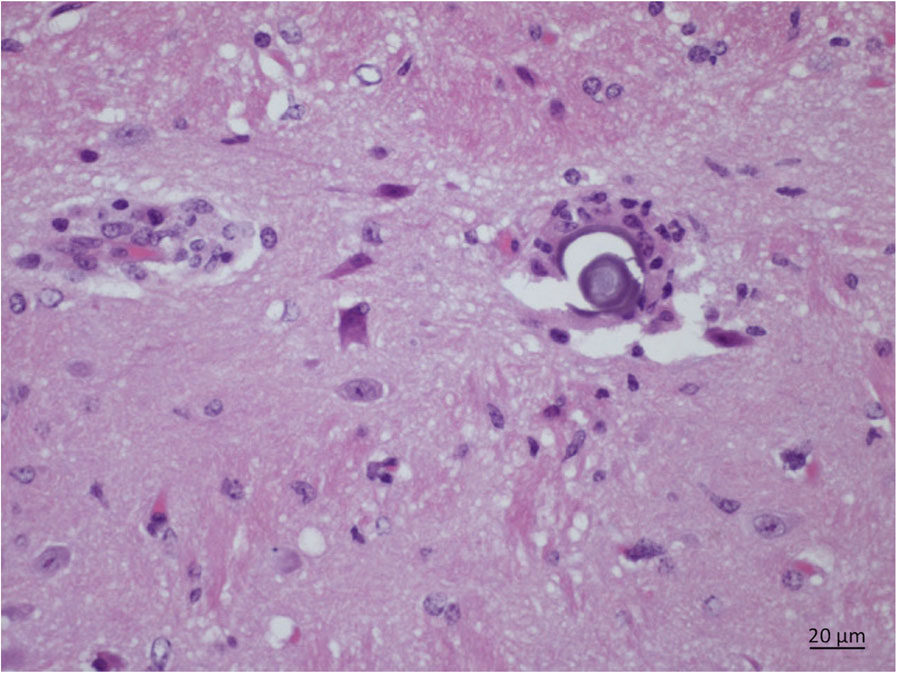
Study 1. Localization of a third-stage larva of *Aelurostrongylus abstrusus* in the brain of a mouse analysed at 20 dpi. *Scale-bar*: 20 μm

The main observations in the brain tissue comprised multifocal encephalitis with perivascular cuffing of small lymphocytes (Fig. 1a, b) and multifocal lymphocytic meningitis (Fig. 1b) which were observed at each time point. In addition, glial nodules, either small or large, composed of histiocytic-like cells, lymphocytes and rare multinucleated giant cells (Fig. 1c, d) were observed from 20 dpi on. In one mouse examined at 20 dpi, a third-stage larva was found within the cerebrum tissue (Fig. 2).

**Fig. 3:**
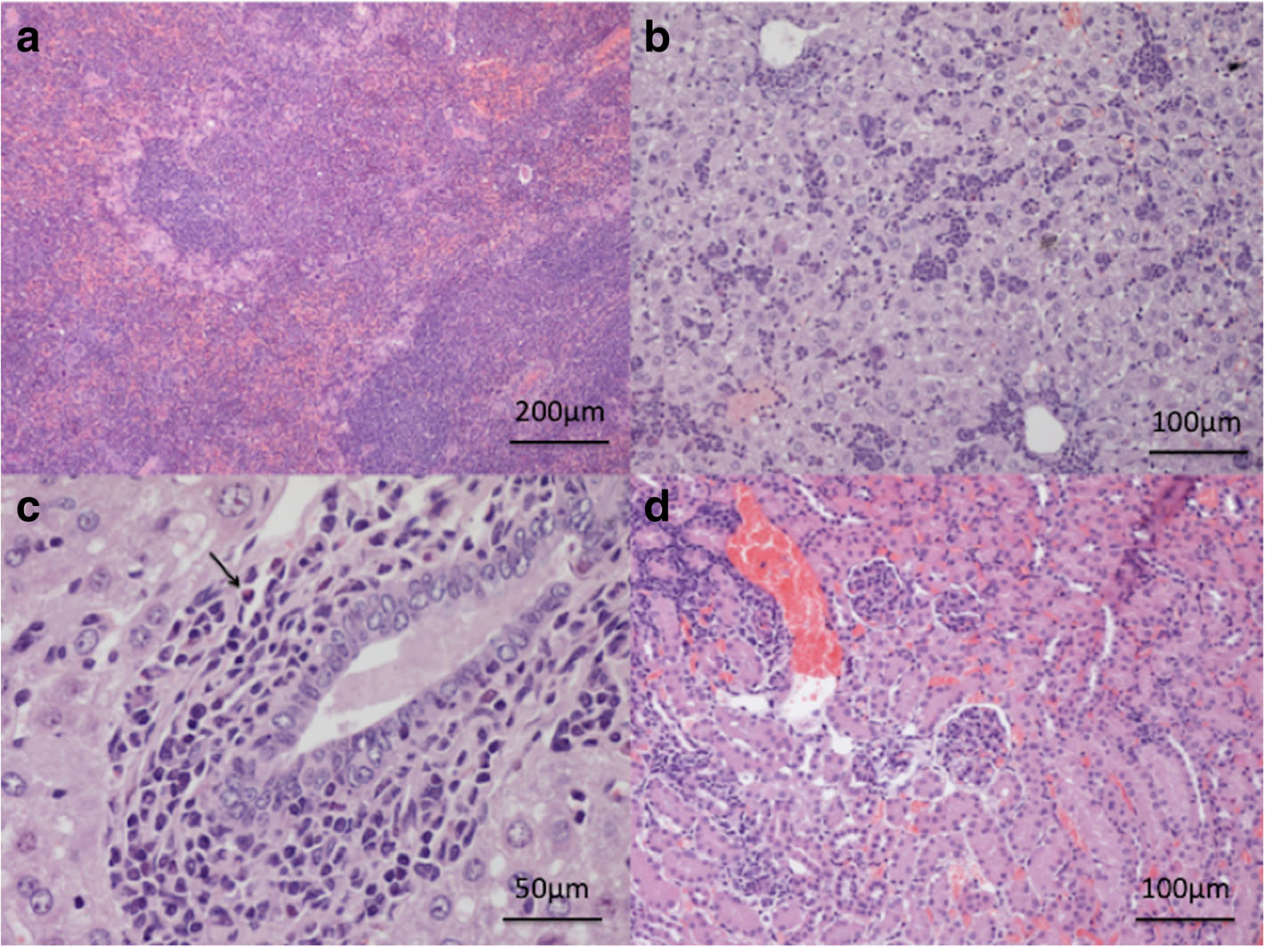
Study 1. **a** Spleen. Hyperplasia with hyalinosis, extramedullary haematopoiesis at 30 dpi. **b** Liver. Severe extamedullary haematopoiesis at 30 dpi. **c** Liver. Cholangitis with very few eosinophils (arrow) at 20 dpi. **d** Kidneys. Interstitial nephritis and glomerulonephritis focal and segmental mesangioproliferative glomerulonephritis at 20 dpi. *Scale-bars*: **a**, 200 μm; **b**, **d**, 100 μm, **c**, 50 μm

Up to 30 dpi, multiple foci of extramedullary haematopoiesis with hyalinosis as a rare finding, and extramedullary haematopoiesis and periductal mixed inflammation were the main changes observed in the spleen and liver tissues, respectively (Fig. 3a, b). In addition, a few eosinophils were detected in periductal location and within small parenchymal granulomas in one mouse examined at 20 dpi (Fig. 3c).

**Fig. 4:**
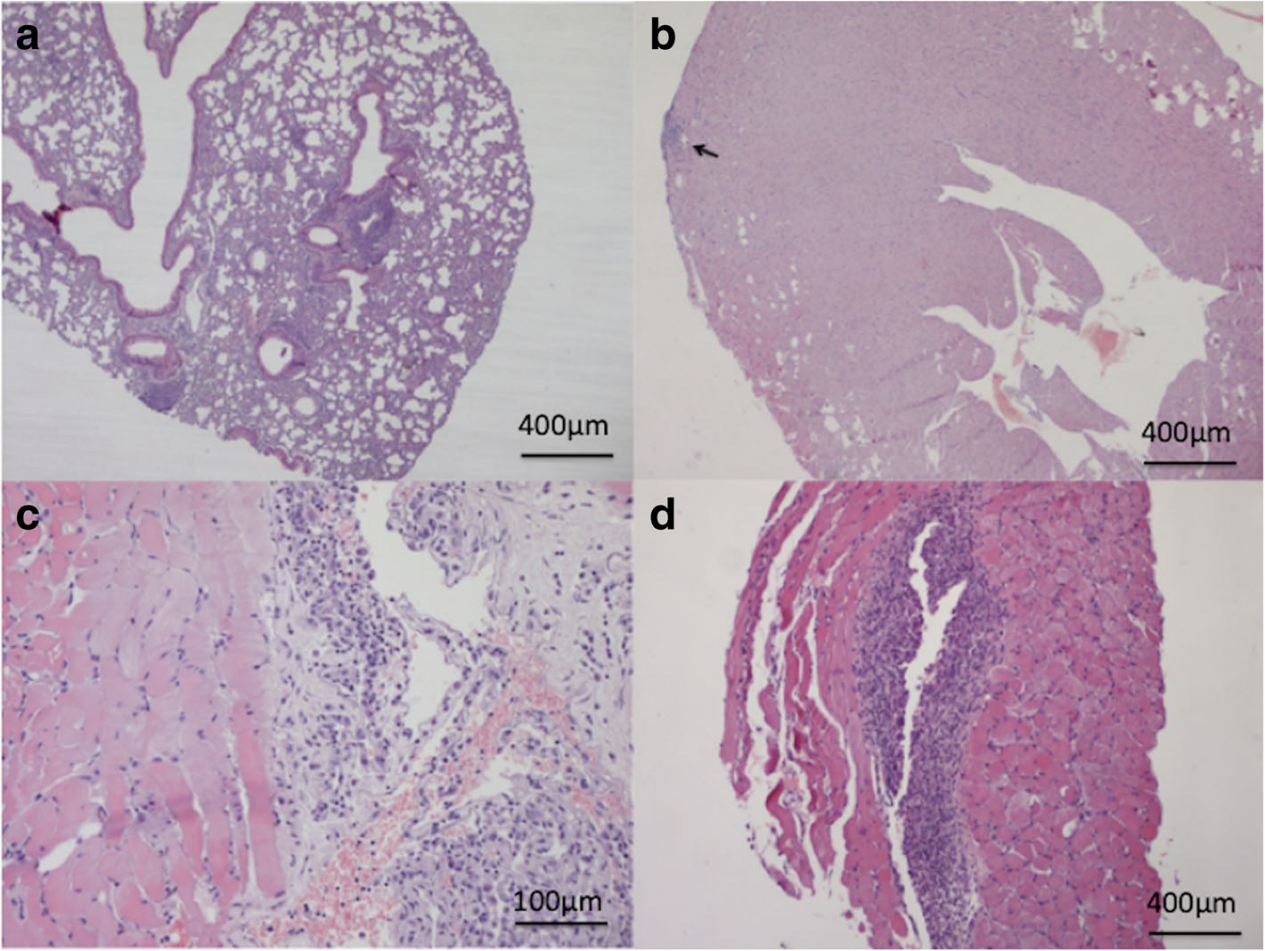
Study 1. **a** Lungs. Peribronchial lymphoid infiltrates at 20 dpi. **b** Heart. Focal very mild subepicardial myocarditis (arrow) at 20 dpi. **c** Diaphragm. Severe granulomatous serositis at 45 dpi. **d** Diaphragm. Granulomatous myositis at 45 dpi. *Scale-bars*: **a**, **b**, **d**, 400 μm; **c**, 100 μm

Multifocal infiltrates (composed mainly of mononuclear cells) in the cortical and medullary interstitium (Fig. 3d) and mild lesions characterized by multifocal lymphocytic peribronchial and occasionally subpleural infiltrates and pneumonitis (Fig. 4a) were the histological changes recorded up to 30 dpi in the kidneys and lungs of infected mice, respectively. Histological examination of the heart was unremarkable, except for one animal which showed a small subepicardial aggregation of lymphocytes at 20 dpi (Fig. 4b). The serous membrane of the diaphragm was markedly thickened through the presence of a dense mixed infiltrate composed of reactive mesothelial cells, macrophages and lymphocytes with rare granulocytes, whereas lymphocytic aggregates were observed within the muscle (diaphragmitis) from 30 dpi on (Fig. 4c, d).

### Study 2

The number of L3 recovered by artificial digestion from the gastrointestinal tract (GIT) organs, internal organs (i.e. combined brain, heart, lungs, spleen and liver) and skeletal muscles of mice each inoculated with 50, 150, 300 or 600 *A. abstrusus* L3 (two mice per dose) and necropsied at 9 dpi, and mean total recovery rates of *A. abstrusus* L3 are shown in Table 3. Mean total recovery rates among these groups ranged from 12% (mice inoculated with 50 *A. abstrusus* L3 each) to 44% (mice inoculated with 600 *A. abstrusus* L3 each). No larvae were isolated from mice of the negative (uninfected) control group. Of the four cats which were fed one mouse each (inoculated with approximately 50, 150, 300 or 600 *A. abstrusus* L3), the two that were fed mice inoculated with 300 and 600 *A. abstrusus* L3 vomited within one hour of feeding; however, the vomit was consumed soon after by the cats. First-stage *A. abstrusus* larvae were recovered from the faeces of all four cats at 43, 44 and 48 dpi (range: 4–910, 2–1050 and 1–5250 larvae per 10 g of faeces, respectively). No larvae were recovered from the faeces of the cat fed with the uninfected mouse.

**Table 3:** Study 2. Number of *A. abstrusus* third-stage larvae (L3) recovered from organs of mice 9 days after inoculation with various numbers of *A. abstrusus* L3 and percentage rate of recovery

No. of L3 inoculated per mouse	No. of L3 recovered from two mice (mean ± standard deviation)				Mean total % recovery rate^c^
	GIT organs	Internal organs^a^	Skeletal muscles	Total^b^	
~50	2 ± 1.4	2 ± 1.4	2 ± 2.8	6 ± 2.8	12
~150	4 ± 5.7	18 ±7.1	14 ± 4.2	36 ± 2.8	24
~300	39 ± 12.7	79 ± 9.9	13.5 ± 9.2	131.5 ± 6.4	44
~600	23 ± 17.0	70 ± 49.5	10 ± 8.5	103 ± 41.0	17

^a^Combined brain, lungs, heart, kidneys, liver, spleen, and diaphragm

^b^Combined GIT organs, internal organs and skeletal muscles

^c^Mean total % recovery rate = 100 × total mean number of *A. abstrusus* L3 recovered/number of *A. abstrusus* L3 inoculated

*Abbreviation*: *GIT* gastrointestinal tract

### Morphological and molecular identification of larvae

Morphology of *A. abstrusus* L1 and L3 collected from the faeces of donor cats or from snails and mice following artificial digestion, respectively, were consistent with previous descriptions [14, 20]. All *18S* rDNA sequences obtained from the L1 and the L3 displayed 100% identity to the nucleotide sequence of *A. abstrusus* deposited in GenBank (accession no. AJ920366).

## Discussion

This study provides information on the localization, histopathological alterations and rate of recovery of *A. abstrusus* L3 in experimentally infected mice and confirms the infectivity of *A. abstrusus* L3-infected mice to cats. *Aelurostongylus abstrusus* L3 were recovered from several organs of mice including the brain, GIT and skeletal muscles, where the majority of larvae were recovered by tissue digestion. Similarly, in previous studies, L3 were mainly found in the organs such as the liver and the omentum of experimentally and naturally infected mice [15, 18]. Yet, a study of localization of larvae at different time points and using various diagnostic techniques (i.e. molecular, histopathology and digestion procedures) was not previously performed. Lesions of various extent were detected by histopathology. Tissue alterations, which featured in the form of infiltrates of lymphocytes, eosinophils and macrophages, granulomas and by glial nodules in the brain, were likely a consequence of *A. abstrusus* larval migration. In the brain, the evaluation of serial longitudinal sections allowed for the description of focal lesions and the detection of one larva at 20 dpi despite the overall low level of challenge with 30 *A. abstrusus* L3. This finding is in accordance with the results of PCR and artificial digestion of tissues which allowed for the detection of *A. abstrusus* DNA and the recovery of *A. abstrusus* L3 from the brain and other organs of mice. The detection of *A. abstrusus* L3 in the brain of mice may be suggestive of neurotropism as shown for other species within the family Angiostrongylidae in other than definitive hosts [1, 21–23]. Whether this anatomical localization influences the behaviour of mice is unknown and was not evaluated in the present studies. However, none of the infected mice in these studies showed signs of behavioural abnormality.

Cats experimentally infected with *A. abstrusus* L3 either within gastropods or through another source invariably vomited [18]. The mechanism causing vomiting is not known, but it is likely that a large number of infective L3 invading the gastric mucosa may cause an irritation, eventually resulting in this outcome [18]. Similarly, the two cats fed with mice that were inoculated with the highest numbers of *A. abstrusus* L3 (i.e. 300 and 600 L3) in Study 2 vomited after consumption of the mice carcasses. Nonetheless, as also observed in these animals, cats may easily feed on vomited material, therefore favouring the *A. abstrusus* transmission.

Mice live in close vicinity to cats, being also present in human dwellings, and represent a much more attractive prey to cats compared to gastropods [12]. Cameron [17, 24] in 1926 claimed the successful development of *A. abstrusus* L1 into L3 in mice and recovered adult nematodes in cats after experimental infection with these mice. However, the length of the L3 (*c.*700 μm) recovered from mouse tissues [17] and the recovery of adult nematodes from the cardiopulmonary blood vessels of experimentally infected cats [14, 18, 25] remained puzzling. Therefore, it was argued that an as-yet-unknown species of lungworm infected the cats [18]. Although no species assignation will be possible for Cameron’s findings, *Angiostrongylus chabaudi* is the only metastrongyloid residing in the vessels of the cardiopulmonary system of felids with L3 measuring approximately 700 μm in length [8]. Since cardiopulmonary nematodes may infect both domestic and wild cats and display a complementary transmission pattern in their life-cycles [8], studies on the biology of lungworm species should be encouraged to better define the epidemiology of metastrongyloids infecting felids [26].

## Conclusions

Although the role of mice in the epidemiology of feline aelurostrongylosis is well recognized, in only one study was a single wild-caught *Apodemus* mouse found to harbour *A. abstrusus* L3 [15]. Data presented here add knowledge to further understand the biology of *A. abstrusus* in mice and emphasize the risk for the infection of cats living in *A. abstrusus* endemic areas. Further studies should be performed to assess the risk factors related to the presence of mice in the epidemiology of feline aelurostrongylosis.

## Methods

### Study design

Two studies were designed to document histopathological tissue lesions in mice experimentally infected with *A. abstrusus* L3 (Study 1), and to determine *A. abstrusus* L3 counts in the organs of infected mice and the infectivity in cats after ingestion of *A. abstrusus-* infected mice (Study 2).

### Infection of snails and recovery of *A. abstrusus* L3

Field isolates of *A. abstrusus* from naturally infected cats from Italy (Study 1) or Hungary (Study 2) were used to infect snails (*Cornu aspersum*). Both isolates had been passed through snails and cats (donor cats) for at least 3 years in the laboratory before being used in these studies. Two days unfed snails were individually placed in infection chambers exposed to *A. abstrusus* L1 as previously described [27]. Twenty-one days from the exposure to larvae, snails were humanely euthanized by menthol steam exposure in a plastic box and the snail tissues were subjected to an artificial digestion protocol to recover *A. abstrusus* L3s [27]. The sediment was washed twice and the larvae were collected in a clean solution with the aid of a micropipette under a light microscope. The product was considered the bulk L3 suspension.

### Experimental infection of mice

In total 30 male mice (*Mus musculus*) weighing 12.4–16.7 g were used (Study 1, 15 mice; Study 2, 15 mice). The mice were acclimatized to the study environment for at least seven days prior to inoculation. Individual doses of *A. abstrusus* L3 (i.e. Study 1, approximately 30 L3; Study 2, approximately 50, 150, 300 and 600 L3) were prepared and suspended in saline solution. For inoculation, the individual inoculum was concentrated to < 0.05 ml, withdrawn in a 1 ml disposable syringe and administered orally by means of a buttoned cannula. Care was taken to ensure complete swallowing of the inoculum.

### Study 1

Ten mice were inoculated with approximately 30 *A. abstrusus* L3 each. At 10, 20, 30, 45 and 60 dpi two mice (designated as ʻMouse 1ʼ and ʻMouse 2ʼ), were randomly selected from the group, humanely euthanized and necropsied. The remaining five mice served as uninfected controls, and one of these was processed in parallel at each day of necropsy.

At each time point the brain, lungs, heart, kidneys, liver, spleen and diaphragm of the ʻMouse 1ʼ were split into two equal portions of which one was subjected to artificial peptic digestion as described in [27] and the other portion was processed for the histopathological examination. In the ʻMouse 2ʼ, the abovementioned organs were similarly split into two portions and processed either for histopathology or molecular analysis.

For histopathological examination, organs were fixed in 10% buffered formalin solution (pH 7.4), and embedded in paraffin. Sections of 5 μm were stained with haematoxylin and eosin. To ensure an adequate histological evaluation, samples were obtained according to the guideline for sampling and trimming in mice [28, 29]. In particular, the following anatomical sections of organs were evaluated: brain (forebrain at level of the optic chiasm, at the base of the posterior hypothalamus and at the level of the caudal thalamus, mid cerebellum and medulla oblongata), spleen (transverse section), liver (left lateral and right medial lobe), kidneys (longitudinal section), lung (left lobe), heart (longitudinal section) and diaphragm (longitudinal section). All tissue sections were carefully read for pathological changes. Organs of the control mice were only subjected to examination for gross lesions and to digestion procedures.

### Study 2

Fifteen mice were formed randomly in five groups of three, and three mice each were inoculated with approximately 50, 150, 300 or 600 *A. abstrusus* L3 as described above or remained uninfected as negative control. At 9 dpi all mice were humanely euthanized.

Two mice of each group were necropsied and the GIT organs (stomach and intestine including mesentery and omentum), internal organs (combined brain, lungs, heart, kidneys, liver, spleen and diaphragm) and skeletal muscles were collected and subjected to artificial digestion. Following digestion, the number of *A. abstrusus* L3 was counted, and the mean total % recovery rate [(number of L3 collected from the organs’ digest/number of inoculated L3) × 100] was calculated.

The carcass of one mouse of each group was fed to one European Short Hair cat (male castrated, purpose bred, approximately 11 to 14 months of age) that was confirmed negative for *A. abstrusus* based on faecal examination prior to inoculation. For inoculation of the cats, carcasses of mice were cut in pieces and offered to the cats in place of their regular food. Care was taken that the carcass was completely consumed. Individual 10 g faecal samples were collected from all cats at 43, 44 and 48 dpi and subjected to the Baermann-Wetzel migration technique for *A. abstrusus* L1 recovery and count.

### Morphological and molecular identification of nematode larvae

*Aelurostrongylus abstrusus* L1 and L3 were identified based on their morphology according to previous descriptions [14, 20]. For molecular analysis, 10 L1 collected from the faeces of the donor cats and 10 L3 collected after artificial digestion of snail tissues were used. Genomic DNA was extracted using a commercial kit (DNeasy Blood & Tissue Kit; Qiagen, Hilden, Germany) from the larvae and selected organs of the experimentally infected mice, in accordance with the manufacturer’s instructions, and a partial fragment of the nuclear *18S* rDNA (~1700 bp) ribosomal gene was amplified using the primers NC18SF1 (5'-AAA GAT TAA GCC ATG CA-3') and NC5BR (5'-GCA GGT TCA CCT ACA GAT-3'), as previously described [20]. The amplicons were purified and sequenced in both directions using the same primers as for PCR, employing the Taq Dye Deoxy Terminator Cycle Sequencing Kit v.2 (Applied Biosystems, Foster City, California, USA) in an automated sequencer (ABI-PRISM 377; Applied Biosystems, Foster City, California, USA). Sequences were edited and aligned using the Geneious R9.1 software package (http://www.geneious.com) and compared with those available in the GenBank database, using the Basic Local Alignment Search Tool (BLAST, http://blast.ncbi.nlm.nih.gov/Blast.cgi).
